# Phylogenetic Comparison of Swainsonine Biosynthetic Gene Clusters among Fungi

**DOI:** 10.3390/jof8040359

**Published:** 2022-03-31

**Authors:** Marwa Neyaz, Sumanjari Das, Daniel Cook, Rebecca Creamer

**Affiliations:** 1 Plant and Environmental Science Department, New Mexico State University, Las Cruces, NM 88003, USA; marwane@nmsu.edu; 2 Biology Department, New Mexico State University, Las Cruces, NM 88003, USA; sdas@nmsu.edu; 3 Poisonous Plant Research Laboratory, United States Department of Agriculture, Agriculture Research Service, Logan, UT 84341, USA; daniel.cook@usda.gov; 4 Entomology, Plant Pathology, and Weed Science Department, New Mexico State University, Las Cruces, NM 88003, USA

**Keywords:** swainsonine, orthologous gene cluster, phylogeny

## Abstract

Swainsonine is a cytotoxic alkaloid produced by fungi. Genome sequence analyses revealed that these fungi share an orthologous gene cluster, SWN, necessary for swainsonine biosynthesis. To investigate the SWN cluster, the gene sequences and intergenic regions were assessed in organisms containing *swnK*, which is conserved across all fungi that produce swainsonine. The orders of fungi which contained orthologous swainsonine genes included Pleosporales, Onygenales, Hypocreales, Chaetothyriales, Xylariales, Capnodiales, Microthyriales, Caliciales, Patellariales, Eurotiales, and a species of the Leotiomycetes. *SwnK* and *swnH2* genes were conserved across all fungi containing the SWN cluster; in contrast, *swnT* and *swnA* were found in a limited number of fungi containing the SWN cluster. The phylogenetic data suggest that in some orders that the SWN cluster was gained once from a common ancestor while in other orders it was likely gained several times from one or more common ancestors. The data also show that rearrangements and inversions of the SWN cluster happened within a genus as species diverged. Analysis of the intergenic regions revealed different combinations and inversions of open reading frames, as well as absence of genes. These results provide evidence of a complex evolutionary history of the SWN cluster in fungi.

## 1. Introduction

Secondary metabolites are organic molecules produced by plants, bacteria, and fungi and are critical for virulence, defense, and communication. The secondary metabolite swainsonine is a toxic indolizidine alkaloid that inhibits α-mannosidase and disrupts the endomembrane system of the animal cells causing a lysosomal storage disease and inhibiting mannosidase II in the Golgi apparatus altering glycoprotein synthesis [1,2,3]. Swainsonine was first isolated from the plant *Swainsona canescens* (Fabaceae) in Australia [4] and subsequently other plant genera within the Convolvulaceae, Fabaceae, and Malvaceae [5].

Research has demonstrated that all swainsonine-containing plants investigated to date host fungal symbionts responsible for the production of swainsonine [5,6,7,8,9]. Other fungi that produce swainsonine are pathogens of insects, mammals, or plants [10,11,12]. Within plants, the seed-transmitted fungal symbiont *Alternaria* section *Undifilum* (Pleosporales) was identified in the locoweeds *Astragalus* and *Oxytropis* (Fabaceae) [8,13,14]. Continued consumption of these plants by grazing animals causes the neurological disease, “locoism”, characterized by weight loss, lack of muscular coordination, altered behavior, depression, infertility, abortion, birth defects, heart congestive failure, and eventually death [15,16]. Another seed transmitted fungal symbiont belonging to order Chaetothyriales was reported in *Ipomoea carnea* (Convolvulaceae) as a swainsonine producer [7]. Swainsonine is also produced by the plant pathogen *Slafractonia leguminicola* [10,17], the insect pathogen *Metarhizium* spp. [11,12,18], and in dermatophytes of the Arthrodermataceae [12].

To understand the evolution of the swainsonine toxin, the swainsonine gene cluster was partially characterized in four orders of fungi: Hypocreales, Chaetothyriales, Onygenales, Pleosporales, and in an undefined order that is part of the class Leotiomycetes [12]. The cluster consists of seven genes that encode functional proteins: a hybrid nonribosomal peptide synthetase (NRPS) polyketide synthase (PKS) *swnK*, *swnH1* and *swnH2* encoding dioxygenases, *swnR* and *swnN* encoding reductases, *swnT* encoding a transmembrane transporter, and *swnA* which encodes an aromatic amino transferase. *SwnK*, the largest gene in the cluster, encodes a multifunctional protein consisted of five domains (A, T, KS, AT, and SDR). Inactivation of *swnK* in *Metarhizium robertsii* resulted in no detectable swainsonine, demonstrating that this gene is required for synthesis of the toxin [12,19].

Several studies have further characterized the swainsonine gene cluster. The KS domain amongst *Alternaria* swainsonine-producing fungi was compared and phylogenetic results indicated that *swnK*-KS sequences differed slightly between plant pathogen and non-pathogens but was highly conserved among all swainsonine-producing *Alternaria* spp. [20]. Similarly, Creamer et al. [21] illustrated high conservation for 15 of 22 of the PKS, NRPS, and PKS/NRPS genes in the genus *Alternaria* and order Pleosporales through phylogenetic analyses.

Many NRPSs and PKSs (highly reducing PKS, partially reducing PKS, and non-reducing PKS) and hybrid PKS/NRPS have been identified in fungi [22] and have been implicated in the biosynthesis of different secondary metabolites [23] including toxins. NRPS and PKSs are key components of toxin pathways for fungi including HC-toxin from the mazie pathogen *Cochliobolus carbonum*, and AM-toxin from the apple pathogen *Alternaria alternata* [24]. NRPSs are important for antibiotic production, such as penicillin produced by *Penicillium* and *Aspergillus* spp., and the immune suppressive cyclosporine produced by *Tolypocladium inflatum* [25]. The diverse roles and species acquisition of these genes reflect a complicated evolutionary history.

Fungal secondary metabolites may be highly divergent [26]. Recombination, gene gain, whole cluster gain and loss, duplications, domain shuffling, neofunctionalization, and subfunctionalization events of secondary metabolites have been reported, as has horizontal gene transfer (HGT) [27]. Interestingly, Lawrence et al., (2011) [28] suggested that an interkingdom gene transfer of a hybrid NPS/PKS from bacteria to *Ascomycota* had occurred, suggesting a possible bacterial ontogeny. Notably, *swnK* represents one of the hybrid NPS/PKS evolutionary outcomes of this interkingdom gene transfer [12].

The evolution of the swainsonine gene cluster, how it was derived, spread, and diversified is unknown. Herein, we evaluate the evolutionary history of the swainsonine genes cluster through BLAST analyses and a comparative phylogenetic framework. We searched for orthologues of the seven swainsonine genes in diverse fungi, assessed the relationship amongst these fungi, and analyzed the intergenic regions of all seven genes, for this work. Our study provides evidence of a complex evolutionary history of the SWN cluster in fungi.

## 2. Materials and Methods

Protein sequences of SWN gene clusters (SwnA-EXU97977, SwnH1-EXU97978, SwnH2-EXU97983, SwnK-EXU97982, SwnN-EXU97980, SwnR-EXU97981, and SwnT-EXU97979) of the fungus *Metarhizium robertsii* order *Hypocreales* were obtained from NCBI as reference sequences. Homology of each SWN protein component was compared using the pblast program of NCBI. SwnK was used as the reference gene to initiate all analyses. Protein sequences for SwnK were identified first; all 36 organisms that contained SwnK were used for subsequent analyses. The sequences for each SWN protein (if available) were obtained from NCBI, downloaded in fasta file format (Table 1).

The intergenic regions of the swainsonine gene cluster for 14 representative fungal species were obtained from NCBI for *Pyrenophora seminiperda*, *Periconia macrospinosa*, *Alternaria oxytropis*, *Clohesyomyces aquaticus*, *Chaetothyriaceae* sp., *Metarhizium acridum*, *Microsporum canis*, *Nannizzia gypsea*, *Trichophyton mentagrophytes*, *Pseudogymnoascus* sp., *Pseudovirgaria hyperparasitica*, *Rosellinia necatrix*, *Xylaria hypoxylon*, and *Tothia fuscella*. The intergenic regions were analyzed to determine all possible combinations (characters) (Table 2). The genomic positions for each gene in each representative fungus were also recorded (Supplementary Table S1A,B). The intergenic regions were analyzed for possible open reading frames (ORFS) through NCBI ORFS Finder tool. ORFS for each fungus were blasted and ORFS that had significant similarities were recorded (Supplementary Tables S2–S14).

Sequences of each protein were compared with Geneious Prime^®^ 2020.2.2 and aligned with MUSCLE with the following parameters: sequences grouped by similarity, anchor optimization, distance measure kmer6_6, clustering method UPGMB, tree rooting method pseudo, and sequence weighting scheme CLUSTALW. Sequences were manually edited to remove any low-quality bases. Maximum parsimony trees were used to construct the phylogenetic protein trees for the seven swainsonine genes and the five intergenic-region trees using PAUP* plugin with heuristic search strategy, fastStep search type and 1000 replications. The outgroup for each SWN gene represented the closest non-SWN-containing taxon of the Hypocreales. The binary tree was constructed using winclada and nona, max trees 100, and 10 replications. The ITS tree was constructed using nucleotides sequences from NCBI for the 14 representative species: *Pseudovirgaria hyperparasitica* (EU041767.1), *Pseudogymnoascus* sp. (MN417288.1), *Chaetothyriales* sp. (MW222228.1), *Tothia fuscella* (MH865619.1), *Periconia macrospinosa* (JX427048.1), *Pyrenophora seminiperda* (MW577415.1), *Alternaria oxytropis* (KJ143732.1), *Xylaria hypoxylon* (GU300095.1), *Rosellinia necatrixisolate* (EF026117.1), *Clohesyomyces aquaticus* (MT627678.1), *Trichophyton mentagrophytes* (KT253558.1), *Nannizzia gypsea* (MT328584.1), *Microsporum canis* (EU200371.1), and *Metarhizium acridum* (HM055449.1), and *Tothia* was the outgroup. *Slafractonia* was not included in this analysis because the SWN cluster is not contiguous. Trees were built using PHYML 3.3.20180621 [29], substitution model HKY85, and X1000 bootstrap values.

An isolate of *Pyrenophora seminiperda* was obtained from Susan Meyer of the U.S. Forest Service in Provo, UT. The isolate was grown on PDA for 14 days. Mycelia was air dried and subsequently analyzed for swainsonine. Swainsonine was detected using methods previously described [30,31].

## 3. Results

### 3.1. Swn Genes

All swainsonine gene matches were to fungal species, representing 11 orders within the Ascomycota, with the exception of *Quercus suber*. *Quercus suber* belongs to the kingdom Plantae. *SwnK* and *swnH2* genes were identified from all fungi assessed in this study, while *swnA* and *swnT* were the identified for the fewest fungi (Table 1). The *swnA* gene was the least frequently identified among the fungi assessed and was not identified among all Pleosporales and Xylariales members, as well as the *Fusarium* sp., *Tothia fuscella*, *Pseudogymnoascus* sp., and *Rhizodiscinia lignyota*. *Quercus suber* contained all the SWN genes with the exception of *swnR*, *swnT*, and *swnA*.

**Figure 1:**
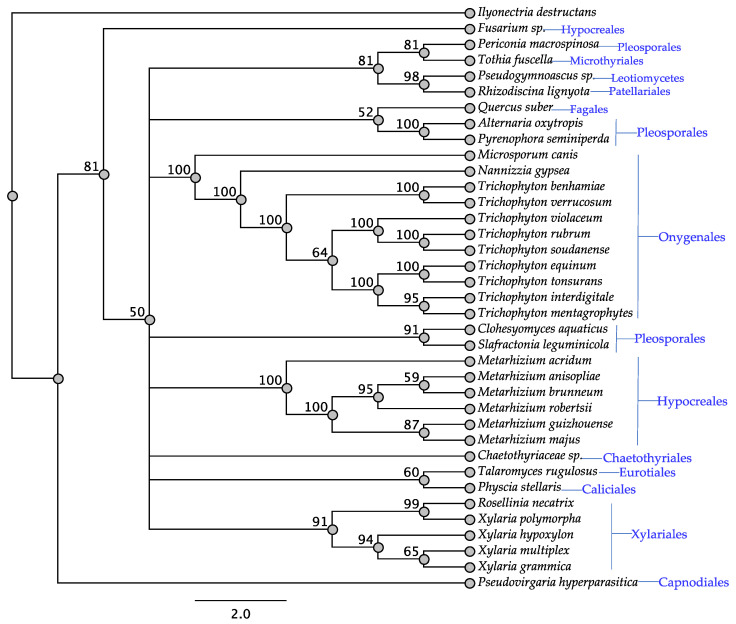
Maximum parsimony protein tree resulting from the analysis of SwnK. Bootstrap confidence values from 1000 bootstrap replicates are presented at each corresponding node. *Ilyonectria destructans* (Hypocreales) was used as the outgroup and obtained from NCBI database, GenBank accession: KAH7007558.1. Fungal orders are indicated next to each organism/clade.

As expected, the *swnK* gene was identified in all the fungi assessed (Table 1, Figure 1) since it was used as the basis for inclusion in the study. Protein similarity, as shown in the SwnK tree, was generally shared by fungi within the same order, with the exception of the *Fusarium* sp. from the Hypocreales and the taxa from the Pleosporales which formed three different clades. Notably, there was a clade of four taxa representing at least three orders with bootstrap support of 81%. All taxa that have been reported to contain swainsonine contained *swnK* consistent with reports showing it to be essential for swainsonine biosynthesis [12] (Table 1).

**Figure 2:**
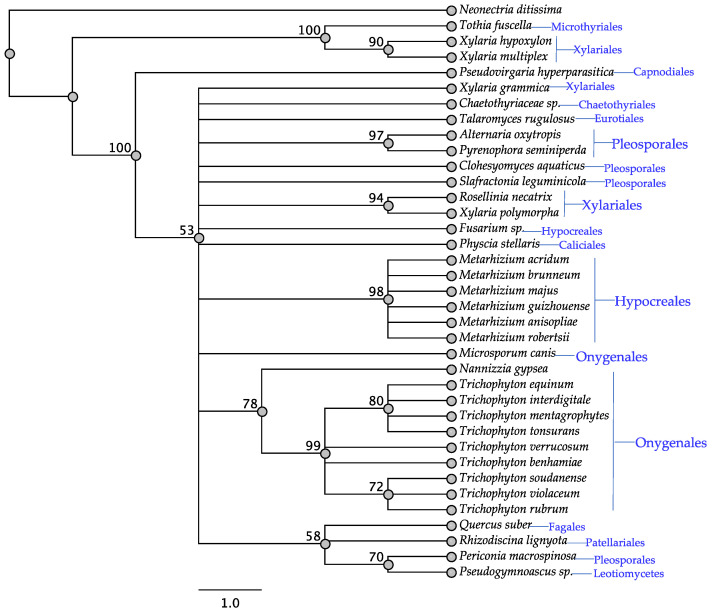
Maximum parsimony protein tree resulting from the analysis of SwnH2. Bootstrap confidence values from 1000 bootstrap replicates are presented at each corresponding node. *Neonectria ditissima* (Hypocreales) was used as the outgroup and obtained from NCBI database, GenBank accession: KPM44118.1. Fungal orders are indicated next to each organism/clade.

All taxa that have been reported to contain swainsonine contained *swnH2* as did all taxa that contained *swnK* (Table 1). The SwnH2 tree was similar to SwnK for the fungal orders, Hypocreales and Onygenales, with the exception of *Microsporum canis* not grouping together with other Onygenales taxa. Other members from other orders diverged in the SwnH2 tree as well (Figure 2), including some taxa of the Xylariales and Pleosporales. For example, in the Xylariales two taxa grouped together with 94% bootstrap support while two others grouped with a taxon from the Microthyriales with 100% bootstrap support.

**Figure 3:**
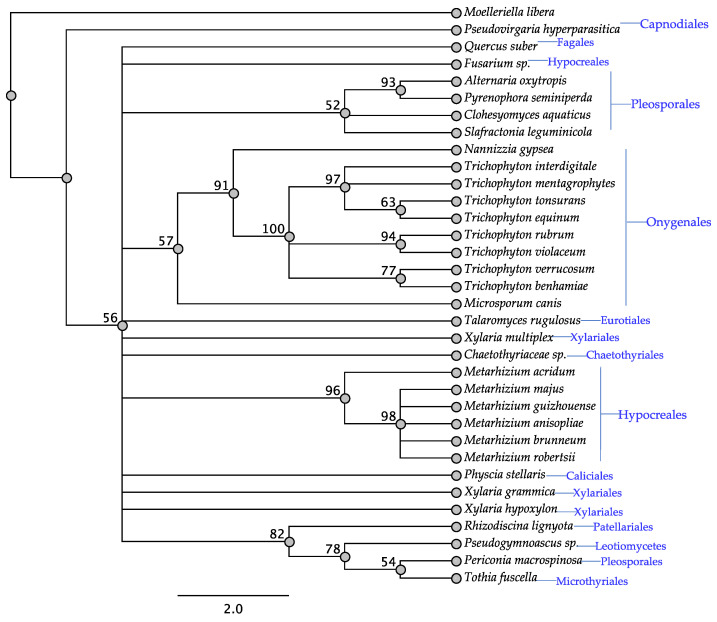
Maximum parsimony protein tree resulting from the analysis of SwnN. Bootstrap confidence values from 1000 bootstrap replicates are presented at each corresponding node. *Moelleriella libera* (Hypocreales) was used as the outgroup and obtained from NCBI database, GenBank accession: KZZ92942.1. Fungal orders are indicated next to each organism/clade.

The swnN gene was found in all taxa of interest except *Rosellinia necatrix* and Xylaria polymorpha (Table 1). All taxa that have been reported to contain swainsonine contain *swnN* (Table 1). The SwnN tree was similar to the SwnK tree for the order Onygenales and Hypocreales. The SwnN tree was unique in that all of the Pleosporales members with the exception of *Periconia macrospinosa* grouped together with 52% bootsrap support, which was not observed in the other trees (Figure 3). Similar to the SwnK tree, there was the clade of four taxa representing at least three orders with a bootstrap support of 82%.

**Figure 4:**
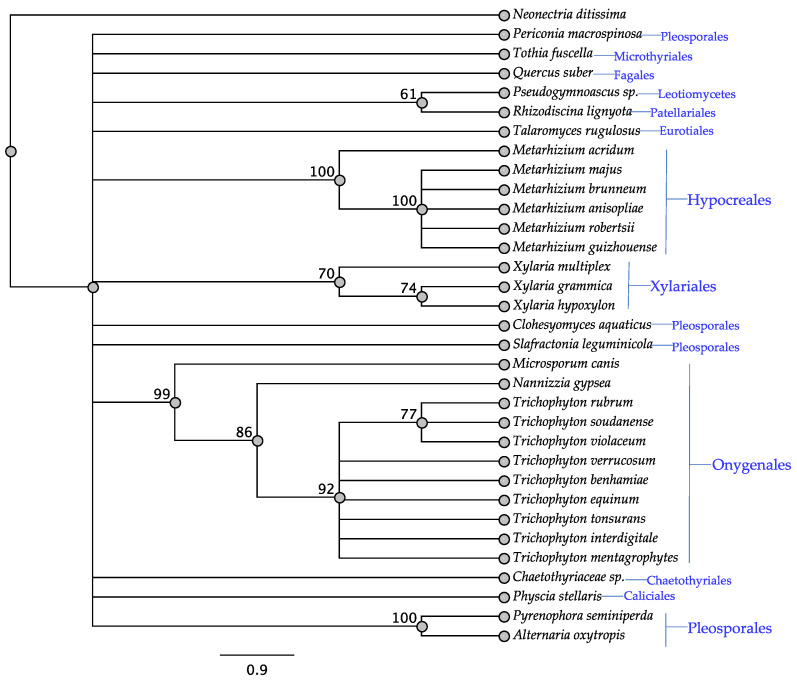
Maximum parsimony protein tree resulting from the analysis of SwnH1. Bootstrap confidence values from 1000 bootstrap replicates are presented at each corresponding node. *Neonectria ditissima* (Hypocreales) was used as the outgroup and obtained from NCBI database, GenBank accession: KPM44118.1. Fungal orders are indicated next to each organism/clade.

The swnH1 gene was found in all fungi except for *Rosellinia necatrix*, *Xylaria polymorpha*, and *Pseudovirgaria hyperparasitica* (Table 1). All taxa that have been reported to contain swainsonine contain *swnH1* (Table 1). The SwnH1 tree showed a similar pattern to the SwnK tree where similar members of the Hypocreales, Onygenales, and Xylariales, and some Pleosporales grouped together (Figure 4).

**Figure 5:**
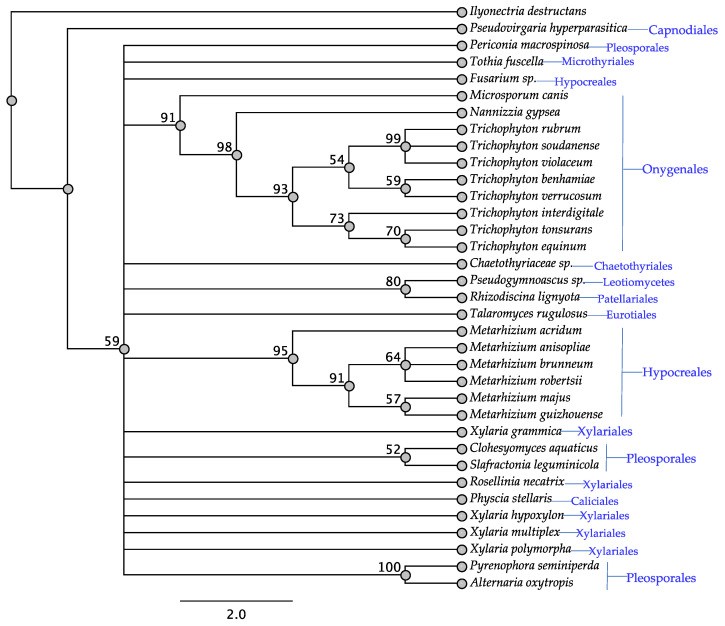
Maximum parsimony protein tree resulting from the analysis of SwnR. Bootstrap confidence values from 1000 bootstrap replicates are presented at each corresponding node. *Ilyonectria destructans* (Hypocreales) was used as the outgroup and obtained from NCBI database, GenBank accession: KAH7007559.1. Fungal orders are indicated next to each organism/clade.

The swnR gene was found in all fungi examined except *Trichophyton mentagrophytes* (Table 1). All taxa that have been reported to contain swainsonine contain swnR (Table 1). In the *SwnR* tree, only the Hypocreales and Onygenales members formed their own clades. Members of the Pleosporales showed a similar grouping pattern to the SwnK tree in which the Pleosporaceae species (*P. seminiperda* and *A. oxytropis*) grouped into one clade, and the *C. aquaticus* and *S. leguminicola* grouped into another clade (Figure 5).

**Figure 6:**
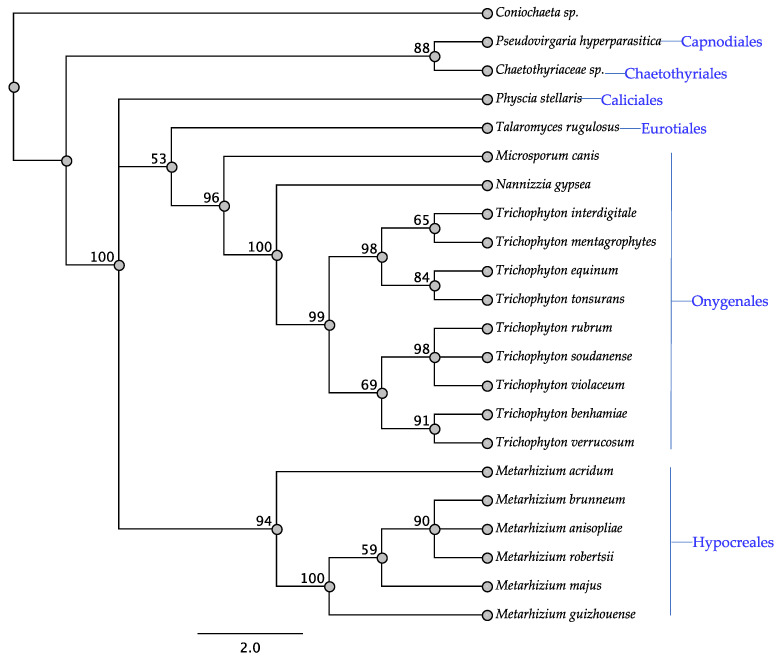
Maximum parsimony protein tree resulting from the analysis of SwnA. Bootstrap confidence values from 1000 bootstrap replicates are presented at each corresponding node. *Coniochaeta* sp. (Hypocreales) was used as the outgroup and obtained from NCBI database, GenBank accession: KAB5525774.1. Fungal orders are indicated next to each organism/clade.

The *swnA* gene was detected in the Onygenales, all Hypocreales with the exception of the *Fusarium* sp., and a single taxon of four other orders (Table 1). Taxa that have been reported to produce and not produce swainsonine were among the taxa that contained *swnA* (Table 1). Members of the Hypocreales and Onygenales grouped together in the SwnA tree, respectively, and were part of a larger clade that included the two taxa belonging to the *Eurotiales* and *Caliciales*, while the two taxa belonging to the Capnodiales and the Chaetothyriales grouped together into a single clade with 88% bootstrap values (Figure 6). Notably, the grouping together of the two taxa belonging to the Capnodiales and the Chaetothyriales had not been observed in other trees.

**Figure 7:**
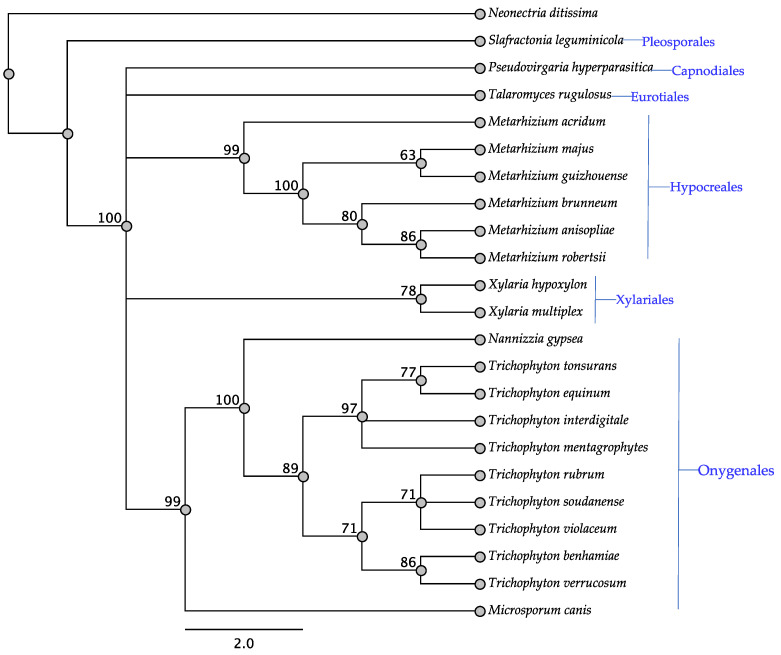
Maximum parsimony protein tree resulting from the analysis of SwnT. Bootstrap confidence values from 1000 bootstrap replicates are presented at each corresponding node. *Neonectria ditissima* (Hypocreales) was used as the outgroup and obtained from NCBI database, GenBank accession: KPM36795.1. Fungal orders are indicated next to each organism/clade.

The *swnT* gene was detected in the Hypocreales, Onygenales, two taxa of the Xylariales, and single taxa of three other orders. Taxa that have been reported to produce and not produce swainsonine were among the taxa that contained *swnT* (Table 1). Interestingly *Slafractonia leguminicola* was the only taxon of the Pleosporales found to contain *swnT* (Table 1, Figure 7). Similar to other trees, in the SwnT tree, members of the Onygenales, Hypocreales, and Xylariales grouped together, respectively (Figure 7).

### 3.2. Intergenic Regions

**Figure 8:**
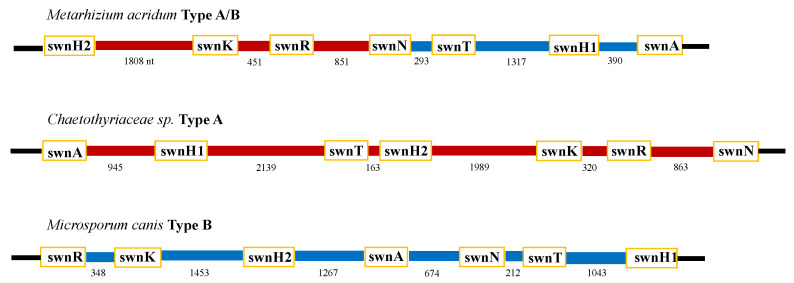
Intergenic region types map of three representative fungi. Intergenic regions colored red indicates type A combination (ex: *swnH2-swnK* in Chaetothyriaaceae) and intergenic regions colored blue indicates type B combination (ex: *swnK–swnH2* in *M. canis*). *Metarhizium acridum* contained both types A and B are labeled as A/B type. Chaetothyriaceae sp. only contained type A (A dominant), and *Microsporum canis* only contained type B (B dominant). Numbers under intergenic regions represent their size, nt = nucleotides.

The intergenic regions between the SWN genes for 14 representative fungi were assembled and analyzed (Table 2). Six fungi with the following 13 characteristics (combinations) were denoted as Type A: *swnH2–swnK*, *swnR–swnN*, *swnK–swnR*, *swnH1–swnT*, *swnN–swnH1*, *swnT–swnN*, *swnT–swnH2*, *swnH2–swnR*, *swnH1–swnH2*, *swnA–swnH2*, *swnA–swnH1*, *swnN–swnA*, and *swnR–swnH1* (Table 2, Figure 8). Inverted intergenic regions were detected in two fungi (except for *swnT–swnH2* and *swnR–swnH1* that had no inversions) were denoted as Type-B. Six fungi had a mix of both types and were classified as Type A/B.

The *swnH2* gene appeared to be the most variable position in that it was found joined with all the other genes, except with *swnN*. The *swnN* and *swnH1* genes were found in four combinations, and *swnR*, *swnT*, and *swnA* genes were found in three combinations. The *swnK* was the least variable and it joined only with *swnH2* and *swnR*.

The sizes of the intergenic regions were greater than 500 bp for most of the fungi with exception to the intergenic region between *swnH1* and *swnH2*, which was under 50 bp in the Pleosporales (Table 2). Intergenic regions greater than 1000 bp were recorded with the hydrogenases: *swnH2–swnK*, *swnH1–swnT*, *swnT–swnH2*, *swnH2–swnR*, *swnA–swnH2*, and *swnR–swnH1*. The intergenic region between *swnH2–swnK* was conserved in all fungi expect for *Clohesyomyces aquaticus*, and the region between *swnR-swnN* was the second most conserved. The Chaetothyriaceae sp. fungus and *Xylaria hypoxylon* possessed the largest intergenic region of *swnH2–swnK* and *swnH1–swnT*. *Alternaria oxytropis* contained a large intergenic region of 2007 bp between *swnN-swnH1*.

The intergenic region between *swnH2–swnK* was shared by all but one fungus assessed, *Clohesyomyces aquaticus* (Table 2). The intergenic regions *swnR–swnN*, *swnK–swnR*, *swnH1–swnT*, *swnN–swnH1*, and *swnT–swnN* were shared by five or more of the 14 fungi assessed. The intergenic regions *swnT–swnH2*, *swnH2–swnR*, *swnH1–swnH2*, *swnA–swnH2*, and *swnN–swnA* were found in three of the fungi assessed. The intergenic region *swnA–swnH1* was found in two fungi, and *swnR–swnH1* was found only in *Periconia macrospinosa*.

**Figure 9:**
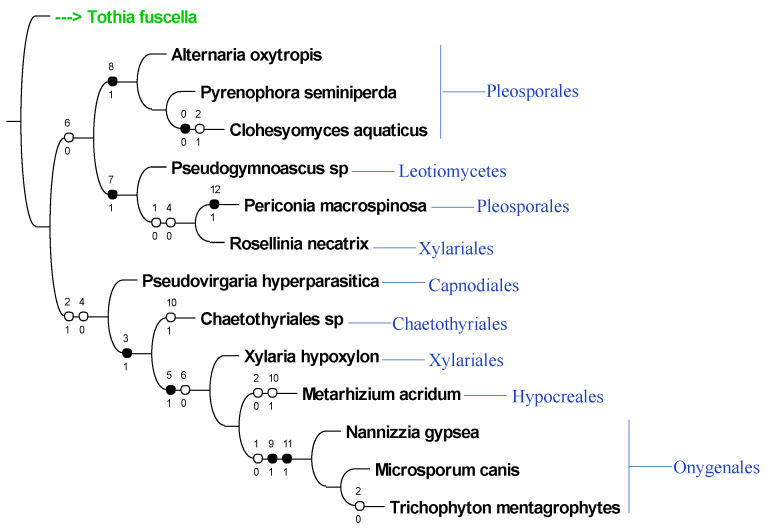
Binary phylogenetic tree of the presence or absence of intergenic region combinations (characters) in representative fungi. The 0–12 numbers on top of each circle represent character number with 0 being assigned to *swnH2*–*swnK* (see Table 2). The 0 and 1 under each circle represent absence (0) or presence (1) of that character. The closed circles indicate that the character only changes at that one spot on the tree, and the open circles indicate changes elsewhere on the tree also.

In the phylogenetic tree of the intergenic regions, the 14 fungi separated into two major groups (Figure 9). One group consisted of all Pleosporales members, *Pseudogymnoascus* sp., and *Rosellinia necatrix*, and the other group consisted of all other fungi. *Alternaria oxytropis*, *Pyrenophora seminiperda*, and *Clohesyomyces aquaticus* were separated from *Periconia macrospinosa* by characters 7 (*swnH2–swR*), 8 (*swnH1–swnH2*), and 12 (*swnR–swnH1*). The black circle on the branches indicates the characters only changed at that spot on the tree, while number 1 indicates the presence of that character and 0 indicates absence. *Xylaria hypoxylon* shared more similarities with the bottom group, and the Onygenales shared the highest number of similarities.

**Figure 10:**
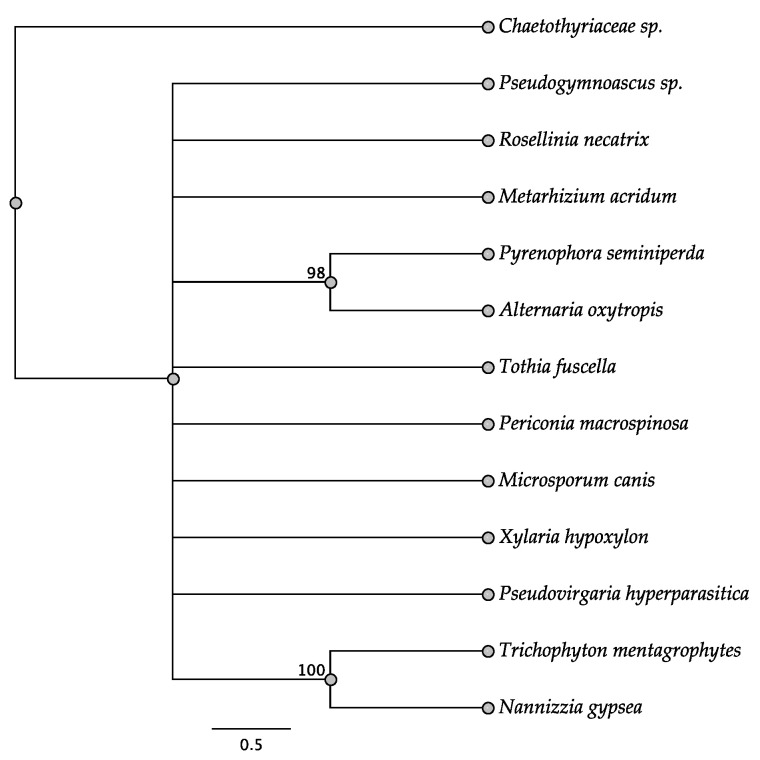
Maximum parsimony DNA tree resulting from the analysis of the *swnH2–swnK* intergenic region sequence data. Bootstrap confidence values from 1000 bootstrap replicates are presented at each corresponding node. Fungal orders are indicated next to each organism/clade.

**Figure 11:**
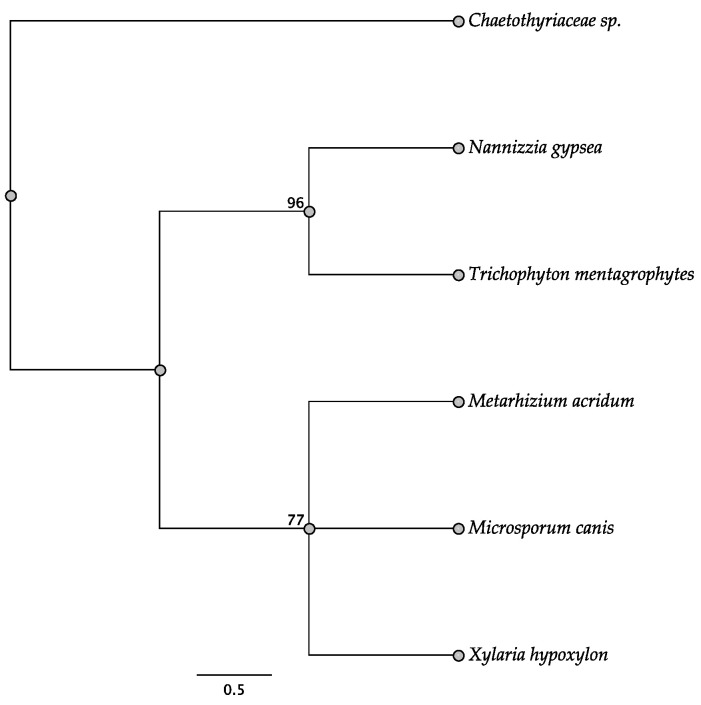
Maximum parsimony DNA tree resulting from the analysis of the *swnH1*–*swnT* intergenic region sequence data. Bootstrap confidence values from 1000 bootstrap replicates are presented at each corresponding node. Fungal orders are indicated next to each organism/clade.

**Figure 12:**
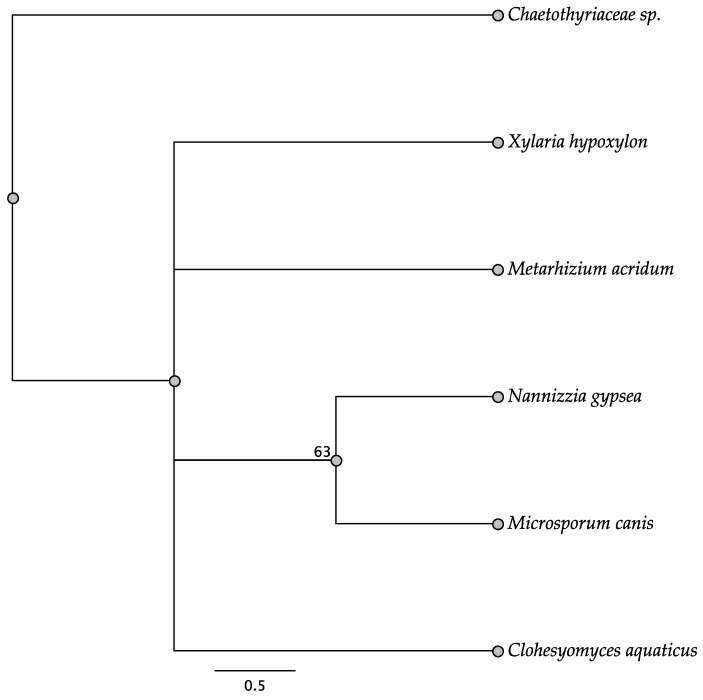
Maximum parsimony DNA tree resulting from the analysis of the *swnK*–*swnR* intergenic region sequence data. Bootstrap confidence values from 1000 bootstrap replicates are presented at each corresponding node. Fungal orders are indicated next to each organism/clade.

**Figure 13:**
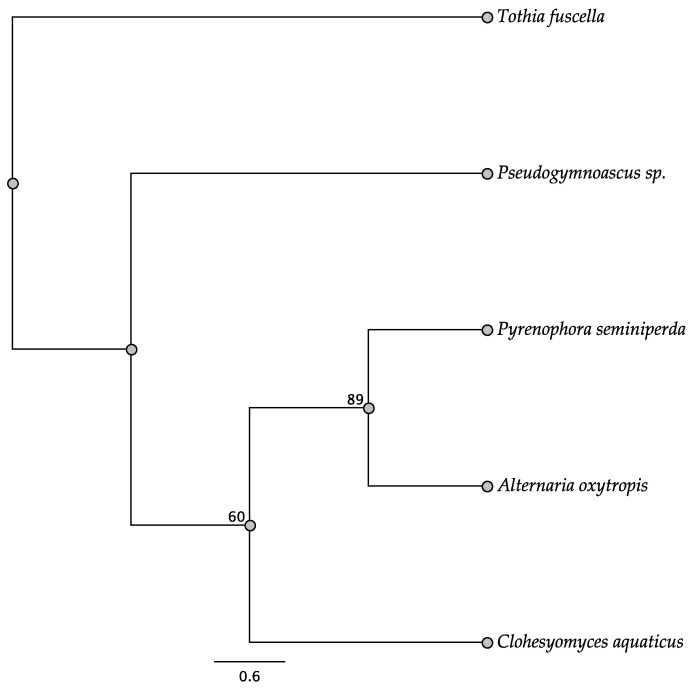
Maximum parsimony DNA tree resulting from the analysis of the *swnN*–*swnH1* intergenic region sequence data. Bootstrap confidence values from 1000 bootstrap replicates are presented at each corresponding node. Fungal orders are indicated next to each organism/clade.

**Figure 14:**
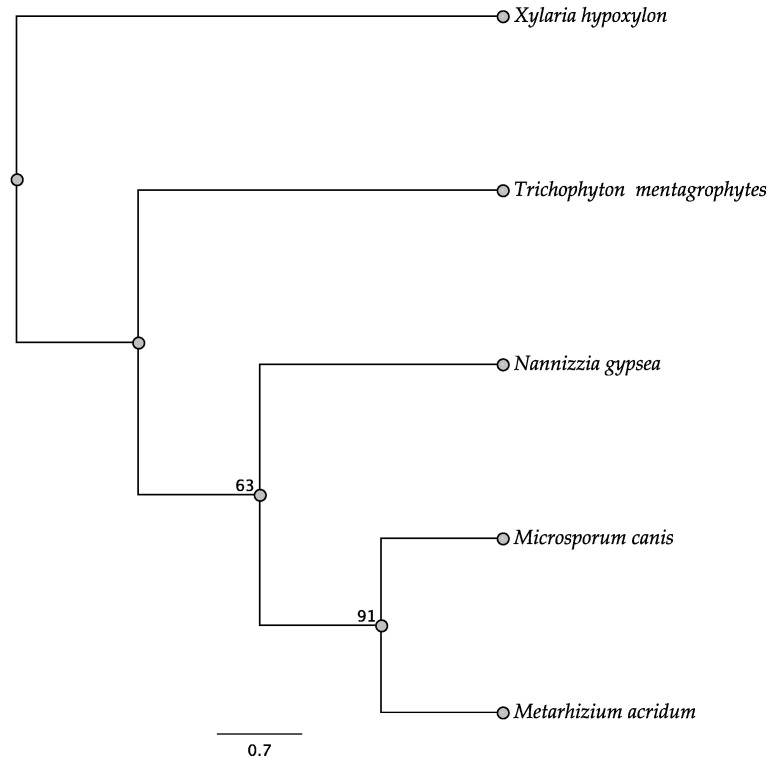
Maximum parsimony DNA tree resulting from the analysis of the *swnT*–*swnN* intergenic region sequence data. Bootstrap confidence values from 1000 bootstrap replicates are presented at each corresponding node. Fungal orders are indicated next to each organism/clade.

Five trees of the intergenic regions were informative among some of the taxa investigated: *swnH2–swnK*, *swnH1–swnT*, *swnK–swnR*, *swnN–swnH1*, and *swnT–swnN*. In the *swnH2–swnK* tree, two members of the Pleosporales, *Alternaria oxytropis*, and *Pyrenophora seminiperda*, and two members of the Onygenales, *Nannizzia gypsea* and *Trichophyton mentagrophytes*, grouped together, respectively, with high boots strap support (Figure 10). These same taxa grouped together with high bootstrap support in other intergenic trees when they were present (Figure 11, Figure 12, Figure 13 and Figure 14). In some trees these respective taxa grouped with other members of the same order, respectively, such as *Microsporum* canis and *Clohesyomyces aquaticus* (Figure 11, Figure 12, Figure 13 and Figure 14).

### 3.3. ITS Phylogeny

**Figure 15:**
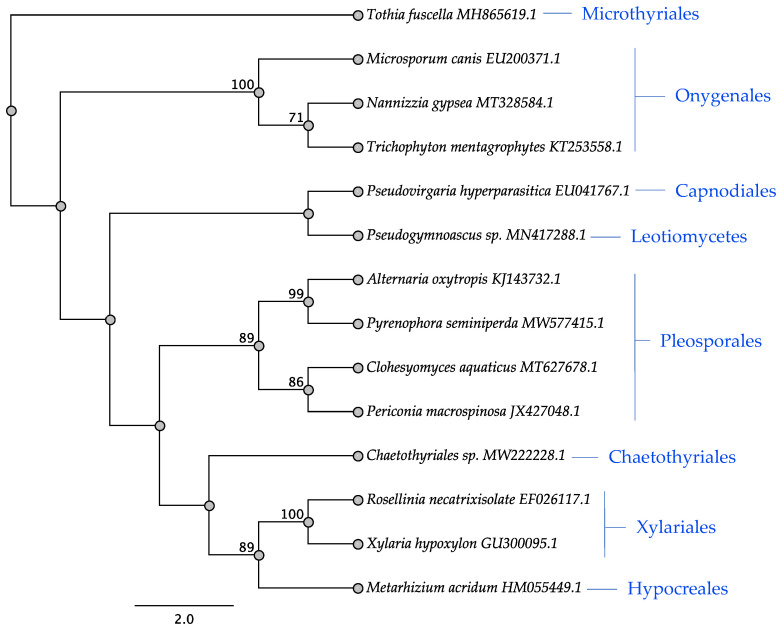
DNA tree resulting from the analysis of the Internal Transcribed Spacer ITS sequence data. Bootstrap confidence values from 1000 bootstrap replicates are presented at each corresponding node. *Tothia fuscella* was used as the outgroup. Fungal orders are indicated next to each organism/clade.

A phylogenetic tree of the internal transcribed sequences (ITS) for the 14 representative fungi was constructed to help compare the relationships between the fungi for this set of noncoding regions with those from the SWN intergenic regions and SWN proteins. Taxa belonging to the same orders, Onygenales, Pleosporales, and Hypocreales grouped together with good boot strap support as one would expect (Figure 15). *Metarhizium acridum* (Hypocreales) grouped with the Xylariales with high bootstrap support consistent with the fact that they both belong to the same fungal class, Sordariomycetes.

### 3.4. Open Reading Frames within the Intergenic Regions

Many of the intergenic regions contained open reading frames (ORFS) (Table 2, Supplementary Tables S2–S14). However, generally only *ORFS* under 30 amino acids (aa) showed matching identity with proteins in databases. Most matches were to fungi, with much fewer matches to bacteria, and amoebae. There were also matches to plants, which may be due to errors in the databases. The swnN–swnH1, swnT–swnH2, and swnR–swnH1 intergenic regions matched only fungi, while the other intergenic regions had matched with bacteria or amoebae also. Overall, 5 of the 14 fungi assessed only matched fungal ORFS.

The fungi *Aspergillus*, *Fusarium*, the Onygenales *Trichophyton benhamiae*, *Metarhizium robertsii*, and *Saccharomyces cerevisiae* were recorded repeatedly as fungal matches (Supplementary Tables S2–S14). Interestingly, the yeast fungus *Saccharomyces cerevisiae* was recorded repeatedly with high percentages, although for non-related functions. Some high fungal percent identity and query cover were observed. For example, the yeast fungus *Cryptococcus neoformans* var. *neoformans* shared 80% query cover and 90% percent identity with *Periconia macrospinosa* in the *swnH2–swnK* intergenic region. Similarly, *Fusarium graminearum* and *Aspergillus nidulans* shared 100% percent identity with *Xylaria hypoxylon* based on their *swnH2–swnK* intergenic region. Other high percentages were recorded with *Aspergillus niger* (*swnH1–swnT*), *Neurospora crassa* (*swnH1–swnT*), *Cryptococcus neoformans* var. *grubii* (*swnN–swnH1*), *Debaryomyces hansenii* (*swnN–swnH1*), *Trichophyton benhamiae* (*swnT–swnH2*, *swnR–swnH1*), *Metarhizium robertsii* (*swnT–swnH2*, *swnR–swnH1*), *Schizosaccharomyces pombe* (*swnN–swnA*), and *Coprinopsis cinerea okayama* (*swnN–swnA*).

Interestingly, some of the ORFS were matches to bacterial species. *Nannizzia gypsea* recorded the highest number of bacterial similarities in the following intergenic regions and their bacterial matches: *swnK–swnR* (*Euplotes aediculatus*), *swnH1–swnT* (*Shewanella piezotolerans*), *swnT–swnN* (*Escherichia coli*), and *swnN–swnA* (*Escherichia coli* and *Agrobacterium tumefaciens*) and percent identity and query cover were high. *Alternaria oxytropis* recorded bacterial similarities as well, in *swnH2–swnK* (*Geobacillus thermodenitrificans*). Other bacterial similarities were homofermentative species *Lacticaseibacillus paracasei* and *Leuconostoc mesenteroides* were matches to *Rosellinia necatrix* in the *swnH2–swnK* with 100% identity and 67% query cover. The Gram-negative fish bacteria *Yersinia ruckeri* was a match to *Clohesomyces aquaticus* in the *swnN-swnR* with 81% query cover and 69.23% identity.

The amoeboid slime mold *Dictyostelium discoideumn* was recorded in *swnH2–swnK* as a match to *Pyrenophora seminiperda*, *Periconia macrospinosa*, and *Pseudogymnoascus* sp., in *swnR–swnN* as a match to Chaetothyriaceae sp., in *swnK–swnR* as a match to Nannizzia gypsea, and in *swnH2–swnA* as a match to *Microsporum canis*. The Xylariales member *Xylaria hypoxylon* shared similarities with the protozoan pathogen *Trypanosoma cruzi* with 80% query cover and 67% identity in the *swnK–swnR*. Four different accessions of *Ipomoea carnea* (Kingdom: Planta) were matches to the seed transmitted fungus Chaetothyriaceae with 100% query covers in *swnH2–swnK*.

Some fungal matches shared predicted functions for their orfs, while others were unrelated to secondary metabolites. The matches to *Fusarium*, *Alternaria*, *Aschochyta*, *Aspergillus*, *Omphalotus*, *Metarhizium*, and *Trichophyton*, showed a function of non-ribosomal peptide synthetase, highly reducing polyketide synthase, non-reducing reducing polyketide synthase, and swainsonine genes. Other fungal matches showed dissimilarities in function, for example, *Saccharomyces cerevisiae* match in the *swnH2–swnK* was a ribonuclease P protein component in the mitochondria. Bacterial matches were more dissimilar in their function.

### 3.5. Swainsonine

Swainsonine was detected in the isolate of Pyrenophora seminiperda (Table 1). Swainsonine was previously detected in several of the species herein [12].

## 4. Discussion

The SWN gene clusters were identified from fungi within the orders Hypocreales, Chaetothyriales, Onygenales, Pleosporales, and a Leotiomycetes sp., as previously reported [12], and in additional species of these orders. Additional orders of fungi were also found to contain the SWN gene cluster including the Xylariales, Capnodiales, Microthyriales, Caliciales, Patellariales, and Eurotiales. The SWN cluster was also detected in the higher plant *Quercus suber* which is not expected as swainsonine has not been shown to be a plant product. We suspect that the *Quercus suber* plant material that was the source of the DNA sequenced was likely associated with a fungal symbiont of some unknown genus that contained the SWN genes. Notably, the morning glory plant *Ipomoea carnea* was matched with the fungus Chaetothyriaceae sp. intergenic region; Chaetothyriaceae sp. is a seed transmitted fungus of the plant *Ipomoea carnea* [7,32]. In both cases, we suspect that these genes belonged to fungi living on or within the plants.

The *swnK* gene was identified in all fungi. This was expected since *swnK* is required for swainsonine production and is highly conserved among swainsonine-producing fungi [12,20]. The *swnH2* gene was found in all taxa also suggesting the important role of this gene for the synthesis of swainsonine. Deletion of the *swnH2* or *swnH1* gene in *Metarhizium robertsii* resulted in the inability of the fungus to produce swainsonine while deletion of *swnN*, *swnR*, *swnT*, and *swnA* reduced swainsonine production to varying amounts in *M. robertsii* but did not eliminate it [19]. These results may suggest that species lacking the SwnH1 gene may not produce swainsonine. Furthermore, not all SWN genes are likely required for swainsonine biosynthesis in all taxa as *Slafractonia leguminicola*, *Pyrenophora seminiperda*, and *Alternaria oxytropis* were missing some of the SWN genes and still produced swainsonine.

In general, the SWN proteins from different fungi that clustered together with the most confidence were also fungi that are the most closely related. For example, all *Trichophyton* and *Metarhizium* species grouped together with greater than 90% percent bootstrap support for each respective protein within the SWN cluster. These results suggest that the SWN gene cluster was present in a recent common ancestor from each of these respective genera. Subsequently as the individual species in these two genera have diverged there have been rearrangements and recombination events that have resulted in the order of the respective SWN genes, as shown herein.

In contrast, the SWN proteins in the different taxa of the Pleosporales clustered with one other Pleosporales species but not others or clustered with taxa representing other orders. Specifically, *Alternaria oxytropis* and *Pyrenophora seminiperda* grouped together in all trees with bootstrap confidence values over 91%, while *Slafractonia leguminicola* and *Clohesyomyces aquaticus* grouped together in the SwnK, SwnR, and SwnN trees. *Pyrenophora macrospinosa* did not group with any member of the Pleosporales, but instead grouped with *Tothia fuscella* (Microthyriales), *Pseudogymnoascus* sp. (Leotiomycetes), and *Rhizodiscinia lignyota* (Patellariales). These results suggest that *Alternaria oxytropis* and *Pyrenophora seminiperda* likely shared the same common ancestor containing the SWN cluster. These results are consistent with a recent report of Creamer et al. [21] demonstrating that several polyketide synthases in *Alternaria oxytropis* were most closely related to paralogs in *Pyrenophora seminiperda* rather than other *Alternaria* species. Further supporting this observation is the fact that none of the closely related *Alternaria* species that have been sequenced contain the SWN cluster. The three groups among the different species of the Pleosporales belong to different families, which might explain the high variability within this group. The patterns observed in this study, combinations and inversions, and absence of genes, suggest that the swainsonine gene cluster was not transferred as a whole-cluster gain to a common ancestor within the Pleosporales but may have originated several times within the order.

*Slafractonia leguminicola* was the only member of the Pleosporales which contained *swnT*. It is likely that SwnT serves a role in transferring swainsonine within or out of the cell as was suggested in Cook et al. [12]. *Slafractonia* was also separated from other fungi in the swnT tree. The presence and absence of different SWN genes among different members of the Pleosporales suggests that the SWN genes may have been inherited from different common ancestors, and that the SWN genes may still be undergoing evolutionary changes within the Pleosporales.

In summary, the divergence of the secondary metabolites gene clusters between closely related Ascomycota spp. is hypothesized to be the result of functional diversity, de novo assembly, and/or horizontal gene transfer [33]. Manning et al. [34] reported a horizontal gene transfer and gene duplications events in NRPSs of *Pyrenophora tritici-repentis*. Horizontal gene transfer and orthologous functional diversity were also discussed as contributing factors to the variability found in *Alternaria oxytropis* SWN genes [21]. In this study, the diversity found within fungal members and the bacterial matches in the ORFs could be due to a combination of horizontal gene transfer, gene duplications, and functional diversity. The high percentage identity recorded for bacterial matches in the open reading frames could suggest bacterial ontogeny. The gene transfer of secondary metabolites from bacteria to ascomycetes was suggested by Lawrence et al. [28], which reported that the hybrid NPS/PKS could have been acquired by Ascomycota via HGT from a bacterial donor in the Burkholderiales early in the evolution of Pezizomycotina. The work here reinforces the diversity and the complex evolution of the swainsonine gene cluster in fungi.

## 5. Conclusions

Few fungal species are known for their production of the toxic alkaloid swainsonine. Despite their diversity, the SWN gene cluster was identified among several fungi. The diversity of the SWN cluster and the intergenic regions within the cluster mirrors the diversity of the various fungal orders; Pleosporales are highly diverse, while the Onygenales and Hypocreales are extremely conserved. The open reading frames in the intergenic regions matched primarily fungi, however the high percentage identity recorded for bacterial matches could suggest bacterial ontogeny for portions of the cluster. These SWN cluster analyses provide a basis for understanding the evolution of secondary metabolites and the mechanisms responsible for the complexity of this cluster in fungi.

## Figures and Tables

**Table 1:** SWN protein homologues and Genebank accession numbers. (-) indicates the absence of that gene in the NCBI database. (*) indicates that the organism has been reported to produce swainsonine (Cook et al., 2017).

Classification		Genebank Accessions						
Genus and Species	Order	swnK	swnH2	swnN	swnH1	swnR	swnA	swnT
*Pyrenophora seminiperda* *	Pleosporales	RMZ73569	RMZ73568	RMZ73566	RMZ73567	RMZ73565	-	-
*Periconia macrospinosa*	Pleosporales	PVI03268	PVI03269	PVI03272	PVI03273	PVI03271	-	-
*Slafractonia leguminicola* *	Pleosporales	AQV04236	AQV04235	AQV04233	AQV04234	AQV04232	-	AQV04231
*Alternaria oxytropis* *	Pleosporales	AQV04230	AQV04229	AQV04227	AQV04228	AQV04226	-	-
*Clohesyomyces aquaticus*	Pleosporales	ORY11783	ORY11779	ORY11781	ORY11780	ORY11782	-	-
*Microsporum canis*	Onygenales	XP_002850891	XP_002850892	XP_002850894	XP_002850896	XP_002850890	XP_002850893	XP_002850895
*Nannizzia gypsea* *	Onygenales	XP_003176907	XP_003176906	XP_003176904	XP_003176902	XP_003176908	XP_003176905	XP_003176903
*Trichophyton benhamiae* *	Onygenales	XP_003014124	XP_003014123	DAA76506	XP_003014119	XP_003016302	XP_003014122	XP_003014120
*Trichophyton verrucosum*	Onygenales	XP_003020763	XP_003020762	XP_003020760	XP_003020758	XP_003022179	XP_003020761	XP_003020759
*Trichophyton violaceum*	Onygenales	OAL75151	OAL75150	OAL75148	OAL75146	OAL69278	OAL75149	OAL75147
*Trichophyton soudanense*	Onygenales	EZF69148	EZF69147	EZF69143	EZF69142	EZF69504	EZF69146	EZF69144
*Trichophyton rubrum* * *118892*	Onygenales	XP_003238870	XP_003238869	XP_003238867	XP_003238865	XP_003238559	XP_003238868	XP_003238866
*Trichophyton tonsurans*	Onygenales	EGD97139	EGD97140	EGD97142	EGD97144	EGD97826	EGD97141	EGD97143
*Trichophyton equinum* *	Onygenales	EGE01982	EGE01983	EGE01985	EGE01987	EGE02341	EGE01984	EGE01986
*Trichophyton interdigitale* * *H6*	Onygenales	EZF30502	EZF30503	EZF30348	EZF30351	EZF32734	KDB22224	EZF30349
*Trichophyton mentagrophytes*	Onygenales	GBF63673	GBF63672	GBF63670	GBF63668	-	GBF63671	GBF63669
*Metarhizium acridum CQM*	Hypocreales	XP_007815889	XP_007815888	XP_007815891	XP_007815893	XP_007815890	XP_007815894	XP_007815892
*Metarhizium anisopliae 53293*	Hypocreales	KJK74452	KJK74453	KJK74450	KJK74448	KJK74451	KJK74447	KJK74449
*Metarhizium brunneum* * *3297*	Hypocreales	XP_014543166	XP_014543167	XP_014543164	XP_014543162	XP_014543165	XP_014543161	XP_014543163
*Metarhizium guizhouense* * *977*	Hypocreales	KID83603	KID83604	KID83601	KID83599	KID83602	KID83598	KID83600
*Metarhizium majus* * *297*	Hypocreales	KID96318	KID96317	KID96320	KID96322	KID96319	KID96323	KID96321
*Metarhizium robertsii* * *23*	Hypocreales	XP_007824811	XP_007824812	XP_007824809	XP_007824807	XP_007824810	XP_007824806	XP_007824808
*Fusarium* sp. NRRL 66182	Hypocreales	KAF5022960	KAF5022961	KAF5022958	KAF5022962	KAF5022959	-	-
*Chaetothyriaceae* sp. *	Chaetothyriales	AQV04224	AQV04220	AQV04221	AQV04219	AQV04222	AQV04223	-
*Xylaria hypoxylon*	Xylariales	TGJ83472	TGJ83466	TGJ83449	TGJ83451	TGJ83448	-	TGJ83450
*Xylaria multiplex*	Xylariales	KAF2965339	KAF2965340	KAF2965337	KAF2965359	KAF2965338	-	KAF2965358
*Xylaria grammica*	Xylariales	RWA14357	RWA14350	RWA14377	RWA14352	RWA14351	-	-
*Xylaria polymorpha*	Xylariales	KAH8158131	KAH8158129	-	-	KAH8158130	-	-
*Rosellinia necatrix*	Xylariales	GAP93000	GAP93001	-	-	GAP93002	-	-
*Pseudovirgaria hyperparasitica*	Capnodiales	XP_033604919	XP_033604918	XP_033604921	-	XP_033604920	XP_033604917	XP_033604916
*Tothia fuscella*	Microthyriales	KAF2430781	KAF2430780	KAF2430777	KAF2430776	KAF2430778	-	-
*Pseudogymnoascus* sp.	Leotiomycetes	KFY51099	KFY51100	KFY51103	KFY51104	KFY51102	-	-
*Quercus suber*	Fagales	XP_023929147	XP_023929150	XP_023929148	XP_023929149	-	-	-
*Physcia stellaris*	Caliciales	KAG6993713	KAG6993712	KAG6993710	KAG6993709	KAG6993711	KAG6993708	-
*Rhizodiscinia lignyota*	Patellariales	KAF2103738	KAF2103739	KAF2103742	KAF2103743	KAF2103741	-	-
*Talaromyces rugulosus*	Eurotiales	XP_035344740	XP_035344739	XP_035344742	XP_035344744	XP_035344741	XP_035344738	XP_035344743

**Table 2:** Intergenic regions (IG) of swainsonine genes. These 13 combinations of IG are represented as characters 0–12. The letters A, B, and A/B placed next to each organism represent the type of combination. Type A are those from 0–12, type B is their inversion, and type A/B indicates the organism has mix of A and B types. Numbers represent IG length. (*) after IG size indicates that IG was inverted (type B). Codes under each IG length (with the letter O) represent open reading frames (ORF). The number before the “O” indicates the number of ORFs with significant matches, and the letters after the “O” represent match type, F = fungus, B = bacteria, and A = amoeba. Other codes: (-) = absence of character, (-----) = blast search did not record any matches, (−v) = overlap, (N/A) = ORFS do not exist. (1O-) no significant matches due to low query cover QC and/or percent identity PI.

Character	0	1	2	3	4	5	6	7	8	9	10	11	12
Organism	swnH2-swnK	swnR-swnN	swnK-swnR	swnH1-swnT	swnN-swnH1	swnT-swnN	swnT-swnH2	swnH2-swnR	swnH1-swnH2	swnA-swnH2	swnA-swnH1	swnN-swnA	swnR-swnH1
*Pyrenophora seminiperda* A	1162 3O/FA	40 -----	-	-	606 2O/F	-	-	-	3 -----	-	-	-	-
*Periconia macrospinosa* A/B	913 * 4O/FA	-	-	-	-	-	-	1808 1O/-	-	-	-	-	1993 6O/F
*Alternaria oxytropis* A	1094 2O/FB	275 1O/A	-	-	2007 1O/F	-	-	-	47 -----	-	-	-	-
*Clohesyomyces aquaticus* B	-	285 2O/FB	651 3O/F	-	511 1O/F	-	-	-	45 -----	-	-	-	-
*Microsporum canis* B	1453 1O/F	-	348 1O/-	1043 -----	-	212 N/A	-	-	-	1267 1O/FA	-	674 1O/F	-
*Nannizzia* *gypsea* A	1774 1O/-	-	621 2O/FA	1080 3O/FB	-	316 2O/B	-	-	-	976 1O/F	-	823 3O/FB	-
*Trichophyton mentagrophytes* A	1353 1O/-	-	-	1014 4O/F	-	306 -----	-	-	-	970 3O/F	-	878 -----	-
*Metarhizium acridum* A/B	1808 6O/F	851 1O/-	451 2O/F	1317 * 1O/F	-	293 * -----	-	-	-	-	390 * 1O/-	-	-
*Chaetothyriaceae* sp. A	1989 3O/F	863 3O/FA	320 1O/-	2139 2O/F	-	-	163 -----	-	-	-	945 3O/B	-	-
*Xylaria hypoxylon* A/B	2581 * 3O/F	870 * -----	184 * 3O/F	1576 1O/-	-	842 1O/F	-	-	-	-	-	-	-
*Rosellinia necatrix* A/B	1664 2O/B	-	-	-	-	-	-	1208 * 1O/A	-	-	-	-	-
*Pseudovirgaria hyperparasitica* A	899 1O/F	154 -----	−v	-	-	-	1910 6O/F	-	-	-	-	-	-
*Tothia* *fuscella* A/B	437 3O/F	−v *	-	-	805 * 2O/F	-	1646 3O/F	-	-	-	-	-	-
*Pseudogymnoascus* sp. A/B	1519 * 6O/FA	568 2O/F	-	-	639 1O/F	-	-	1958 3O/F	-	-	-	-	-

## Data Availability

Not applicable.

## Supplementary Materials

The following supporting information can be downloaded at: https://www.mdpi.com/article/10.3390/jof8040359/s1. Table S1A: SWN gene arrangements in each fungus; Table S1B: The genomic positions. *swnN* intergenic region was not found in *Periconia macrospinosa* as it is in different scaffold; Table S2: Open reading frames ORFs in the intergenic region *swnH2-swnK* information; Table S3: Open reading frames ORFs in the intergenic region *swnR-swnN* information; Table S4: Open reading frames ORFs in the intergenic region *swnK-swnR* information; Table S5: Open reading frames ORFs in the intergenic region *swnH1-swnT* information; Table S6: Open reading frames ORFs in the intergenic region *swnN-swnH1* information; Table S7: Open reading frames ORFs in the intergenic region *swnT-swnN* information; Table S8: Open reading frames ORFs in the intergenic region *swnT-swnH2* information; Table S9: Open reading frames ORFs in the intergenic region *swnH2-swnR* information; Table S10: Open reading frames ORFs in the intergenic region *swnH1-swnH2* information; Table S11: Open reading frames ORFs in the intergenic region *swnA-swnH2* information; Table S12: Open reading frames ORFs in the intergenic region *swnA-swnH1* information; Table S13: Open reading frames ORFs in the intergenic region *swnN-swnA* information; Table S14: Open reading frames ORFs in the intergenic region *swnR-swnH1* information.
