# Supplementary material for: Phylogenetic Comparison of Swainsonine Biosynthetic Gene Clusters among Fungi
Source: J Fungi (Basel). 2022 Mar 31;8(4):359. doi: 10.3390/jof8040359 (PMC9030584; doi:10.3390/jof8040359)
Supplement: Supplementary file 1 [file jof-08-00359-s001.zip › jof-1644761-supplementary.pdf]

Supplementary Table S1A. SWN gene arrangements in each fungus.

Organism	Genes arrangement
Pyrenophora seminiperda	swnR - swnN - swnH1 - swnH2 - swnK
Periconia macrospinosa	swnK - swnH2 - swnN - swnR - swnH1
Alternaria oxytropis	swnR - swnN - swnH1 - swnH2 - swnK
Clohesyomyces aquaticus	swnH2 - swnH1 - swnN - swnR - swnK
Microsporum canis	swnR - swnK - swnH2 - swnA - swnN - swnT - swnH1
Nannizzia gypsea	swnH1 - swnT - swnN - swnA - swnH2 - swnK - swnR
Trichophyton mentagrophytes	swnH1 - swnT - swnN - swnA - swnH2 - swnK
Metarhizium acridum	swnH2 - swnK - swnR - swnN - swnT - swnH1 - swnA
Chaetothyriales sp.	swnA - swnH1 - swnT - swnH2 - swnK – swnR - swnN
Xylaria hypoxylon	swnH1 - swnT - swnN - swnR - swnK - swnH2
Rosellinia necatrix	swnR - swnH2 - swnK
Pseudovirgaria hyperparasitica	swnT - swnH2 - swnK - swnR - swnN
Tothia fuscella	swnH1 - swnN - swnR - swnT - swnH2 - swnK
Pseudogymnoascus sp.	swnK - swnH2 - swnR - swnN - swnH1

Supplementary Table S1B. The genomic positions. *swnN* intergenic region was not found in *Periconia macrospinosa* as it is in different scaffold.

Organism	Genomic position
Pyrenophora seminiperda	swnR - swnN (958,184 - 958,225)
	swnN - swnH1 (959,625 - 960,232)
	swnH1 - swnH2 (961,128 - 961,130)
	swnH2 - swnK (962,203 - 963,366)
Periconia macrospinosa	swnK - swnH2 (108,402 - 109,316)
	swnH2 - swnR (110,329 - 112,138)
	swnR - swnH1 (113,055 - 115,047)
Alternaria oxytropis	swnR - swnN (9,075 - 9,351)
	swnN - swnH1 (10,514 - 12,522)
	swnH1 - swnH2 (13,508 - 13,554)
	swnH2 - swnK (14,569 - 15,664)
Clohesyomyces aquaticus	swnH2 - swnH1 (13,125-13,171)
	swnH1 - swnN (14,184 -14,696)
	swnN - swnR (15,831-16,117)
	swnR - swnK (17,031-17,683)
Microsporum canis	swnR - swnK (2,721,303 - 2,721,650)
	swnK - swnH2 (2,729,276 - 2,730,729)
	swnH2 - swnA (2,731,743 - 2,733,010)
	swnA - swnN (2,734,492 - 2,735,165)
	swnN - swnT (2,736,301 - 2,736,513)
	swnT - swnH1 (2,738,114 - 2,739,156)
Nannizzia gypsea	swnH1 - swnT (2,667,153 - 2,668,232)
	swnT - swnN (2,669,730 - 2,670,045)
	swnN - swnA (2,671,182 - 2,672,004)
	swnA - swnH2 (2,673,487 - 2,674,462)
	swnH2 - swnK (2,675,460 - 2,677,233)
	swnK - swnR (2,684,929 - 2,685,548)
Trichophyton mentagrophytes	swnH1 - swnT (57,109 - 58,124)
	swnT - swnN (59,620 - 59,927)
	swnN - swnA (61,069 - 61,947)
	swnA - swnH2 (63,428 - 64,413)
	swnH2 - swnK (65,411-66,7632)
Metarhizium acridum	swnH2 - swnK (6492-8300)
	swnK - swnR (15,768-16,213)
	swnR - swnN (17,137-17,987)
	swnN - swnT (19,121-19,413)
	swnT - swnH1 (20,887-22,203)
	swnH1 - swnA (23,193-23,582)
Chaetothyriales sp.	swnA - swnH1 (2,059 - 3,005)
	swnH1 - swnT (4,002 - 6,142)
	swnT - swnH2 (7,446 - 7,610)
	swnH2 - swnK (8,634 - 10,624)
	swnK - swnR (18,126 - 18,447)

Xylaria hypoxylon	swnH1 - swnT (14,494 - 16,072)
	swnT - swnN (17,407 - 18,250)
	swnN - swnR (19,391 - 20,262)
	swnR - swnK (21,179 - 21,364)
	swnK - swnH2 (28,869 - 31,451)
Rosellinia necatrix	swnR - swnH2 (8,774 - 9,983)
	swnH2 - swnK (10,981 - 12,646)
Pseudovirgaria hyperparasitica	swnT - swnH2 (295,954 - 297,863)
	swnH2 - swnK (299,330 - 300,228)
	swnK - swnR (308,178 - 308,178) overlap
	swnR - swnN (309,370 - 309,525)
Tothia fuscella	swnH1 - swnN (78,196 - 79,002)
	swnN - swnR (80,363 - 80,224) overlap
	swnT - swnH2 (81,573 - 83,220)
	swnH2 - swnK (84,767 - 85,203)
Pseudogymnoascus sp.	swnK - swnH2 (9,100 - 1,0620)
	swnH2 - swnR (11,630 - 13,589)
	swnR - swnN (14,506 - 15,075)
	swnN - swnH1 (16,198 - 16,838)

Supplementary Table S2. Open reading frames ORFs in the intergenic region *swnH2-swn*K information. **Type** represents the combination type A or B, ex: *Alternaria oxytropis* has type A combination *swnH2-swnK*, while *Periconia macrospinosa* has type B combination *swnK-swnH2*. **Length** represents the intergenic region size, nt= nucleotides. **ORFs** represent the total number of open reading frames. **Match** represent the number of the open reading frame(s) recorded similarity, followed by its **length**, aa= amino acids. **Similarity** represent significant similarities generally ranged between 60-100%, and under 60% in some cases to point out unique event. **QC** stands for query cover, and **PI** present identity. **Accession** numbers are recorded for more information. **Note: ORFs labeled (-)** illustrates lack of open reading frames. **Match labeled (-)** means no similarities even if ORFS exist. **Similarity labeled (-)** means no significant similarities due to low QC and/or PI. This applies to all tables.

Organism	Type	Length	ORFs	Match	Length	Similarity	Accession	QC	PI	Length
Alternaria oxytropis	H2-K	1094 nt	8	3	26 aa	*Aspergillus nidulans*	Q5B6W0.1	57%	63%	908 nt
						Coccidioides immitis	Q1E6U9.2	57%	63%	932 nt
				4	25 aa	*Geobacillus thermodenitrificans*	A4IKW7.1	68%	67%	1242 nt
Pyrenophora seminiperda	H2-K	1162 nt	10	9	28 aa	-				
				10	27 aa	-				
				2	25 aa	*Dictyostelium discoideum*	P0CE95.1	80%	76%	2492 nt
						Aspergillus nidulans	Q5B7W2.2	84%	67%	843 nt
Periconia macrospinosa	K-H2	913 nt	9	4	29 aa	-				
				8	27 aa	*Dictyostelium discoideum*	Q8T674.1	62%	64%	730 nt
				6	26 aa	*Cryptococcus neoformans*	P0CN46.1	80%	90%	885 nt
				9	26 aa	-				
Chaetothyriales sp.	H2-K	1989 nt	19	2	30 aa	*Aspergillus niger*	G3XUF0.1	76%	86%	4870 nt
				17	28 aa	*Trichophyton benhamiae*	D4AZ24.1	75%	50%	909 nt
				8	27 aa	*Aspergillus nidulans*	Q5ATV8.2	51%	69%	623 nt
						Ipomoea purpurea	P48399.1	100%	45%	352 nt
						Ipomoea trifida	P48402.1	100%	45%	366 nt
						Ipomoea purpurea	P48397.2	100%	45%	396 nt
						Ipomoea cordatotriloba	P48394.1	100%	42%	363 nt
						Ipomoea nil	P48395.1	96%	44%	344 nt
Metarhizium acridum	H2-K	1808 nt	21	1	25 aa	*Penicillium chrysogenum*	Q9Y8G8.1	68%	69%	1587 nt
				2	25 aa	-				
				3	25 aa	*Aspergillus oryzae*	Q2U059.1	64%	64%	391 nt
				4	29 aa	*Trichophyton benhamiae*	D4AP52.1	51%	59%	656 nt
				8	29 aa	*Fusarium graminearum*	I1RN14.1	96%	80%	4841 nt

				6	29 aa	*Saccharomyces cerevisiae*	Q02773.1	66%	73%	1202 nt
Microsporum canis	K-H2	1453 nt	13	7	28 aa	*Trichophyton benhamiae*	D4B478.1	75%	50%	704 nt
Nannizzia gypsea	H2-K	1774 nt	11	1	27 aa	-				
Trichophyton mentagrophytes	H2-K	1353 nt	10	6	28 aa	-				
Pseudogymnoascus sp.	K-H2	1519 nt	15	7	27 aa	*Fusarium graminearum*	Q4IP34.2	67%	52%	1207 nt
				1	30 aa	-				
				10	30 aa	-				
				9	29 aa	*Dictyostelium discoideum*	Q54L90.1	79%	67%	1606 nt
				12	25 aa	*Ustilago maydis*	Q4P937.1	88%	60%	2251 nt
						Saccharomyces cerevisiae	E7LYB2.1	84%	62%	843 nt
						Cryphonectria parasitica	Q8HHD2.1	80%	65%	656 nt
				8	30 aa	-				
Pseudovirgaria hyperparasitica	H2-K	899 nt	5	5	30 aa	*Plasmodium falciparum*	O77374.1	73%	61%	1119 nt
Rosellinia necatrix	H2-K	1664 nt	11	10	27 aa	-				
				2	28 aa	*Lacticaseibacillus paracasei*	Q034X0.1	67%	100%	1199 nt
						Leuconostoc mesenteroides	Q03V60.1	67%	100%	1202 nt
Tothia fuscella	H2-K	437 nt	5	2	29 aa	-				
				5	29 aa	-				
				3	25 aa	*Schizosaccharomyces pombe*	Q09837.1	76%	67%	533 nt
						Alternaria cinerariae	A0A0N7D4P6.1	60%	60%	2398 nt
						Ascochyta fabae	A0A5C1RDA3.1	60%	67%	1886 nt
Xylaria hypoxylon	K-H2	2581 nt	20	18	28 aa	*Fusarium graminearum*	I1RN14.1	89%	42%	4841 nt
				7	28 aa	*Fusarium graminearum*	I1RN14.1	67%	100%	4841 nt
				4	25 aa	*Aspergillus nidulans*	Q00681.2	52%	75%	1559 nt
						Aspergillus nidulans	Q5D6D7.2	64%	100%	5386 nt

Supplementary Table S3. Open reading frames ORFs in the intergenic region *swnR-swnN* information.

Organism	Type	Length	ORFs	Match	Length	Similarity	Accession	QC	PI	Length
Alternaria oxytropis	R-N	275 nt	3	1	25 aa	*Dictyostelium discoideum*	Q55GP8.1	60%	60%	648 nt
Clohesomyces aquaticus	N-R	285 nt	3	1	27 aa	*Saccharomyces cerevisiae*	P30822.1	62%	89%	1084 nt
						Ustilago maydis	Q4P5V8.1	66%	60%	539 nt
				3	27 aa	*Omphalotus olearius*	Q52US9.1	77%	73%	4547 nt
						Yersinia ruckeri	C4ULG3.1	81%	69%	2123 nt
Pyrenophora seminiperda	R-N	40 nt	-							
Chaetothyriales sp.	R-N	863 nt	10	7	29 aa	*Aspergillus terreus*	Q0CF73.1	93%	56%	2737 nt
				8	29 aa	-				
				4	25 aa	*Dictyostelium discoideum*	Q54LE6.1	84%	80%	1460 nt
Metharizium acridum	R-N	851 nt	11	10	28 aa	-				
Pseudogymnoascus sp.	R-N	568 nt	5	2	26 aa	*Saccharomyces cerevisiae*	Q03640.1	80%	62%	1545 nt
				5	25 aa	-				
Pseudovirgaria hyperparasitica	R-N	154 nt	1	-						
Xylaria hypoxylon	N-R	870	8	-						
Tothia fuscella	N-R	overlap								

Supplementary Table S4. Open reading frames ORFs in the intergenic region *swnK-swnR* information.

Organism	Type	Length	ORFs	Match	Length	Similarity	Accession	QC	PI	Length
Chaetothyriales sp.	K-R	320 nt	2	1	30 aa	-				
Clohesyomyces aquaticus	R-K	651 nt	8	5	29aa	-				
				2	25 aa	*Saccharomyces cerevisiae*	Q04924.1	72%	70%	702 nt
				4	25aa	*Aspergillus clavatus*	A1C9M6.1	60%	87%	1389 nt
Metarhizium acridum	K-R	451 nt	3	2	29 aa	-				
				3	27 aa	*Kluyveromyces lactis*	Q6CQ43.1	62%	71%	1502 nt
Microsporum canis	R-K	348 nt	2	1	24 aa	-				
Nannizzia gypsea	K-R	621 nt	3	3	27 aa	*Euplotes aediculatus*	O00939.1	78%	87%	1031 nt
						Dictyostelium discoideum	Q551X9.1	67%	73%	1109 nt
				2	26 aa	*Fusarium verticillioides*	W7MLD7.1	50%	100%	3916 nt
						Metarhizium robertsii	E9F8M3.1	65%	53%	2488 nt
Xylaria hypoxylon	R-K	184 nt	3	2	26 aa	-				
				3	26 aa	*Trypanosoma cruzi*	Q4DCH3.1	80%	67%	1086 nt
				1	25 aa	*Aspergillus nidulans*	Q5BDY8.1	100%	52%	6077 nt

Supplementary Table S5. Open reading frames ORFs in the intergenic region *swnH1-swnT* information.

Organism	Type	Length	ORFs	Match	Length	Similarity	Accession	QC	PI	Length
Chaetothyriales sp.	H1-T	2139 nt	15	4	66 aa	*Metarhizium robertsii*	E9F8M0.2	50%	42%	496 nt
				10	26 aa	*Aspergillus oryzae*	C0STD9.1	46%	87%	785 nt
						Pyrenophora teres	E3S6N7.1	84%	45%	463 nt
Metarhizium acridum	T-H1	1317 nt	12	11	26 aa	*Neurospora crassa*	Q7S454.2	57%	100%	599 nt
Microsporum canis	T-H1	1043 nt	14	-						
Nannizzia gypsea	H1-T	1080 nt	11	1	29 aa	*Saccharomyces cerevisiae*	Q06169.1	68%	64%	523 nt
				6	28 aa	*Shewanella piezotolerans*	B8CUW9.1	53%	100%	807 nt
				9	26 aa	-				
Trichophyton mentagrophytes	H1-T	1014 nt	15	9	29 aa	*Aspergillus niger*	G3Y416.1	65%	100%	537 nt
				1	28 aa	-				
				7	26 aa	*Saccharomyces cerevisiae*	P33301.1	84%	50%	854 nt
				4	25 aa	-				
Xylaria hypoxylon	H1-T	1576 nt	9	7	29 aa	-				

Supplementary Table S6. Open reading frames ORFs in the intergenic region *swnN-swnH1* information.

Organism	Type	Length	ORFs	Match	Length	Similarity	Accession	QC	PI	Length
Alternaria oxytropis	N-H1	2007 nt	6	4	29 aa	*Cryptococcus neoformans*	J9VWU3.1	58%	100%	732 nt
Clohesyomyces aquaticus	H1-N	511 nt	5	2	25 aa	*Schizosaccharomyces*	Q9USR3.1	84%	67%	1877 nt
Pyrenophora seminiperda	N-H1	606 nt	6	2	28 aa	*Debaryomyces hansenii*	Q6BJJ8.2	62%	100%	1147 nt
				4	25 aa	*Alternaria solani*	Q5KTN1.1	24%	100%	509 nt
						Alternaria cinerariae	A0A0N7D4P6.1	48%	67%	2389 nt
Pseudogymnoascus sp.	N-H1	639 nt	6	6	29 aa	*Debaryomyces hansenii*	Q6BYY8.1	89%	60%	481 nt
Tothia fuscella	H1-N	805 nt	8	2	28 aa	*Saccharomyces cerevisiae*	N1PA11.1	60%	86%	616 nt
				3	25 aa	*Metarhizium robertsii*	E9EYB5.1	52%	54%	1032 nt

Supplementary Table S7. Open reading frames ORFs in the intergenic region *swnT-swnN* information.

Organism	Type	Length	ORFs	Match	Length	Similarity	Accession	QC	PI	Length
Metarhizium acridum	N-T	293 nt	4	-						
Microsporum canis	N-T	212 nt	-							
Nannizzia gypsea	T-N	316 nt	2	2	27 aa	*Escherichia coli*	P25907.2	62%	75%	786 nt
				1	26 aa					
Trichophyton mentagrophytes	T-N	306 nt	1	-						
Xylaria hypoxylon	T-N	842 nt	6	4	56 aa	*Metarhizium robertsii*	E9F8M0.2	100%	64%	496 nt
						Trichophyton benhamiae	D4AU27.1	100%	66%	498 nt

Supplementary Table S8. Open reading frames ORFs in the intergenic region *swnT-swnH2* information.

**Organism**	**Type**	**Length**	**ORFs**	**Match**	**Length**	**Similarity**	**Accession**	**QC**	**PI**	**Length**
Chaetothyriales sp.	T-H2	163 nt	1	-						
Pseudovirgaria hyperparasitica	T-H2	1910 nt	14	8	429 aa	*Metarhizium robertsii*	E9F8L8.2	80%	47%	498 nt
						Trichophyton benhamiae	D4AU29.1	86%	45%	493 nt
				11	39 aa	*Trichophyton benhamiae*	D4AU26.1	100%	85%	307 nt
						Metarhizium robertsii	E9F8L9.1	100%	82%	307 nt
				7	30 aa	*Saccharomyces cerevisiae*	Q02979.1	56%	86%	1223 nt
				6	27 aa	-				
				10	26 aa	*Saccharomyces cerevisiae*	P53037.1	80%	54%	1138 nt
				12	25 aa	-				
Tothia fuscella	T-H2	1646 nt	11	11	315 aa	-				
				9	29 aa	*Ustilago maydis*	Q4P8F6.1	79%	45%	1715 nt
				5	27 aa	*Fusarium verticillioides*	W7MLD7.1	55%	86%	3916 nt

Supplementary Table S9. Open reading frames ORFs in the intergenic region *swnH2-swnR* information.

Organism	Type	Length	ORFs	Match	Length	Similarity	Accession	QC	PI	Length
Rosellinia necatrix	R-H2	1208 nt	5	3	29 aa	*Dictyostelium discoideum*	Q54M77.1	65%	67%	1867 nt
Pseudogymnoascus sp.	H2-R	1958 nt	15	8	317 aa	-				
				6	27 aa	-				
				1	25 aa	*Aspergillus terreus*	Q0D1P6.1	76%	60%	531 nt
Periconia macrospinosa	H2-R	1808 nt	18	3	72 aa	-				

Supplementary Table S10. Open reading frames ORFs in the intergenic region *swnH1-swnH2* information. No ORFs were recorded due to short sequence size.

Organism	Type	Length	ORFs	Similarity	Length	Matches	Accession	QC	PI	Length
Pyrenophora siminiperda	H1-H2	3 nt	-							
Clohesyomyces aquaticus	H2-H1	45 nt	-							
Alternaria oxytropis	H1-H2	47 nt	-							

Supplementary Table S11. Open reading frames ORFs in the intergenic region *swnA-swnH2* information.

Organism	Type	Length	ORFs	Match	Length	Similarity	Accession	QC	PI	Length
Trichophyton mentagrophytes	A-H2	970 nt	9	1	31 aa	-				
				8	29 aa	-				
				7	28 aa	*Saccharomyces cerevisiae*	P0C2J3.1	78%	60%	1770 nt
Nannizzia gypsea	A-H2	976 nt	6	3	27 aa	*Saccharomyces cerevisiae*	P38329.1	88%	69%	1120 nt
Microsporum canis	H2-A	1267 nt	6	4	26 aa	*Dictyostelium discoideum*	Q1ZXH8.3	65%	87%	1632 nt
						Saccharomyces cerevisiae	Q08562.1	61%	75%	1619 nt

Supplementary Table S12. Open reading frames ORFs in the intergenic region *swnA-swnH1* information.

Organism	Type	Length	ORFs	Match	Length	Similarity	Accession	QC	PI	Length
Metarhizium acridum	H1-A	390 nt	5	3	29 aa	-				
Chaetothyriales sp.	A-H1	945 nt	7	1	29 aa	*Helicobacter acinonychis*	Q17ZG3.1	73%	75%	921 nt
				3	27 aa	-				
				5	25 aa	-				

Supplementary Table S13. Open reading frames ORFs in the intergenic region *swnN-swnA* information.

Organism	Type	Length	ORFs	Match	Length	Similarity	Accession	QC	PI	Length
Trichophyton mentagrophytes	N-A	878 nt	3	-						
Nannizzia gypsea	N-A	823 nt	6	4	28 aa	*Escherichia coli*	P77468.1	71%	69%	867 nt
						Cordyceps militaris	G3J453.1	67%	62%	2525 nt
				2	26 aa	*Schizosaccharomyces pombe*	Q9P6N9.1	88%	100%	331 nt
						Coprinopsis cinerea okayama	A8NU66.2	55%	100%	1065 nt
				3	25 aa	*Agrobacterium tumefaciens*	P0A3V3.1	64%	89%	755 nt
						Escherichia coli	P29018.3	80%	61%	588 nt
Microsporum canis	A - N	674 nt	6	2	25 aa	*Schizosaccharomyces pombe*	Q6LA55.2	80%	55%	2699 nt

Supplementary Table S14. Open reading frames ORFs in the intergenic region *swnR-swnH1* information.

Organism	Type	Length	ORFs	Match	Length	Similarity	Accession	QC	PI	Length
Periconia macrospinosa	R-H1	1993 nt	17	13	184 aa	*Trichophyton benhamiae*	D4AU28.1	97%	61%	318 nt
						Metarhizium robertsii	E9F8M1.1	97%	58%	341 nt
						Botrytis cinerea	B1GVX5.1	45%	30%	300 nt
				11	73 aa	*Penicillium citrinum*	Q8J0F5.1	49%	39%	2563 nt
				15	33 aa	*Metarhizium robertsii*	E9F8M1.1	96%	81%	341 nt
						Trichophyton benhamiae	D4AU28.1	96%	75%	318 nt
						Botrytis cinerea	B1GVX5.1	87%	46%	300 nt
				1	29 aa	-				
				16	27 aa	-				
				2	27 aa	-				
